# Value of diffusion‐weighted imaging in diagnosis and therapy response assessment of hepatic fungal infection in patients with acute leukemia

**DOI:** 10.1002/iid3.843

**Published:** 2023-04-26

**Authors:** Haoyu Wang, Haitao Yu, Dong Bai, Dan Yao, Yongjun Han, Yichao Shi, Zhiqun Wang

**Affiliations:** ^1^ Department of Radiology Aerospace Center Hospital Beijing China; ^2^ Department of Gastroenterology Aerospace Center Hospital Beijing China

**Keywords:** acute leukemia, diffusion‐weighted imaging, hepatic fungal infection

## Abstract

**Objective:**

To investigate the role of diffusion‐weighted imaging (DWI) for diagnosis and posttreatment assessment of hepatic fungal infection in patients with acute leukemia.

**Methods:**

Patients with acute leukemia and highly suspected hepatic fungal infection were collected in the study. All the patients underwent MRI examination, including initial and follow‐up DWI. The apparent diffusion coefficient (ADC) values of the lesions and the normal liver parenchyma were compared using Student's *t*‐test. The ADC values of the hepatic fungal lesions of pretreatment and posttreatment were compared using paired *t*‐test.

**Results:**

A total of 13 patients with hepatic fungal infections have enrolled this study. Hepatic lesions were rounded or oval shaped, measured from 0.3 to 3 cm in diameter. The lesions showed significantly hyperintense signal on DWI and markedly hypointense signal on the ADC map, reflecting a marked restricted diffusion. The mean ADC values of the lesions were significantly lower than those of normal liver parenchyma (1.08 ± 0.34 × 10^−3^ vs. 1.98 ± 0.12 × 10^−3^ mm^2^/s, *p* < 0.001). After treatment, the mean ADC values of the lesions were significantly increased when comparing with those of pretreatment (1.39 ± 0.29 × 10^−3^ vs. 1.06 ± 0.10 × 10^−3^ mm^2^/s, *p* = .016).

**Conclusion:**

DWI can provide diffusion information of hepatic fungal infection in patients with acute leukemia, which could be taken as a valuable tool for diagnosis and therapy response assessment of these patients.

## Graphical abstract

Diffusion‐weighted imaging (DWI) can provide a diffusion information of hepatic fungal infection. DWI could be a valuable tool for therapy response assessment of acute leukemia.

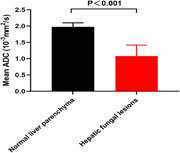

## 1 INTRODUCTION

Hepatic fungal infections occur in immunosuppressed patient, particularly in those with profound and prolonged neutropenia. All sorts of factors include intensive chemotherapy protocols for leukemia, liver transplantation, bone marrow transplantation, prolonged antibiotic therapy, and immunosuppressive therapy, have been suggested as contributors to the increased susceptibility of hepatic fungal infections.^1^, ^2^, ^3^ Importantly, hepatic fungal infection was a serious complication of intensive chemotherapy treatment in patients with acute leukemia,^4^, ^5^ which was associated with high morbidity and mortality. In clinical practice, liver biopsy is often impractical for patients with acute leukemia because of the severity of the clinical condition and the coexistence of other complications, such as thrombocytopenia or disseminated intravascular coagulation. Moreover, a definitive diagnosis of fungal infection in these patients remains challenging because of lacking of specific symptoms, and the findings in specimen cultures or tests are often negative for fungi, which leads to delayed diagnosis and treatment and increased mortality. Therefore, early diagnosis of hepatic fungal infections and prompt antifungal therapy were imperative.

Imaging played a key role in the diagnosis and monitoring response to the treatment of patients with clinically suspected liver infections. Both computed tomography (CT) and ultrasonography were conventional imaging modalities for detection and follow‐up of hepatic fungal infection.^6^, ^7^, ^8^, ^9^ However, Magnetic Resonance Imaging (MRI) was superior to CT and ultrasonography for assessment of hepatic fungal infection, due to its better contrast resolution.^10^ Besides structure analysis, MRI can also provide functional information for diagnosis and therapy response assessment of hepatic diseases. Diffusion‐weighted imaging (DWI) as an MRI functional imaging technique, which has been widely used in abdomen diseases, allowed for the depiction of diffusion features of water molecules. Imaging features of hepatic fungal infection by conventional MRI have been reported in patients with acute leukemia.^11^, ^12^ However, the use of DWI for diagnosis and follow‐up analyses of hepatic fungal infection has not been reported. Thus, this study aims to investigate the value of DWI in the diagnosis and therapy response assessment of hepatic fungal infection in patients with acute leukemia.

## 2 MATERIALS AND METHODS

### 2.1 Patients

**Figure 1:**
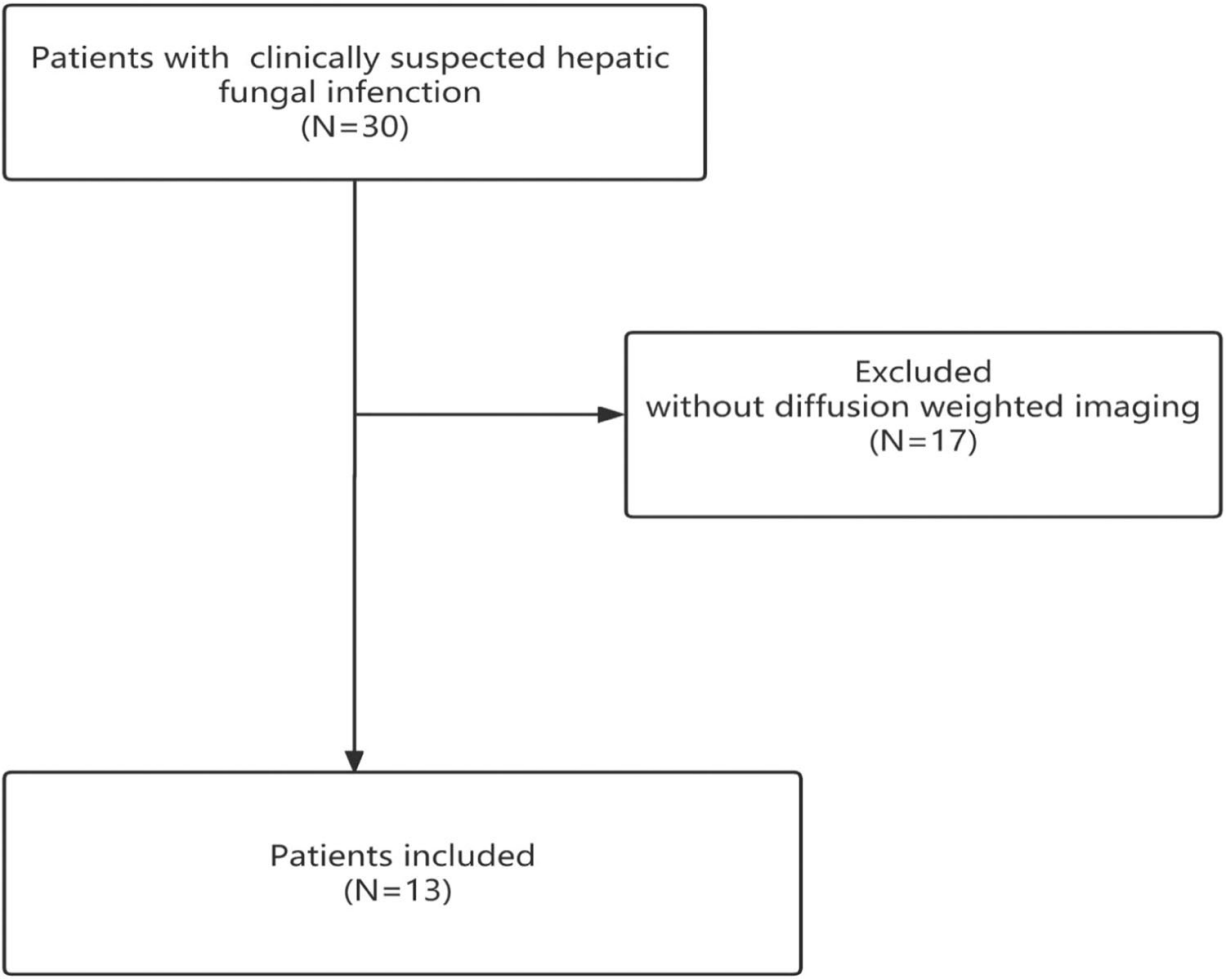
Flow chart of the study.

Our institutional review board approved this study, the study approval number was JHYLS 2022073, informed consent was obtained from the participating patients. Between 2014 and 2021, 30 patients with a clinically suspected hepatic fungal infection, presented persistent fever despite apparently appropriate antibacterial therapy and abdominal pain. All patients underwent conventional MR imaging. However, 17 patients were excluded due to lacking functional DW imaging. Finally, 13 patients with conventional and DW imaging of hepatic fungal infection, performed by chemotherapy or bone marrow transplant due to acute leukemia, were retrospectively analyzed (Figure 1). The diagnosis of hepatic fungal infections was based on the European Organization for Research and Treatment of Cancer and Mycoses Study Group (EORTC‐MSG) guideline.^13^ All the patients with clinically suspected hepatic fungal infection were unresponsive to broad‐spectrum antibiotics, and received the antifungal drugs (voriconazole and amphotericin B) as therapy.

### 2.2 Data collection and analysis

The electronic medical records of patients with hepatic fungal infection were extracted and analyzed by the research team. Clinical manifestations, laboratory data, MRI findings, and treatments were obtained. Information assessed included age, sex, diagnosis of acute leukemia [acute myeloid leukemia (AML) and acute lymphoblastic leukemia (ALL)], symptoms (fever and abdominal pain), laboratory tests (white blood cell count, neutrophil count, alanine aminotransferase and aspartate aminotransferase), and antifungal therapy effect (lesion absorption and disappearance).

### 2.3 MRI acquisition and image analysis

MRI scans were performed using high‐field 3T MRI scanner (Siemens Medical Systems, MAGENTOM). The routine MRI protocol included a breath‐hold T1‐weighted with in‐phase and out‐of‐phase sequences (TR/TE, 4.11/1.23 ms; flip angle, 12°; matrix, 320 × 320; slice thickness, 3 mm; intersection gap, 0.6 mm; field of view, 320 × 260 mm) and a respiratory‐triggered T2‐weighted FSE sequence (TR/TE, 3500/87 ms; flip angle, 94°; fat suppression, matrix, 320 × 320; slice thickness, 5 mm; intersection gap, 1.5 mm; field of view, 320 × 320 mm). A dynamic contrast‐enhanced sequence was performed by using Gd‐DTPA (gadolinium ‐ diethylenetriamine pentaacetic acid) at a dosage of 0.01 mmol/kg during the arterial, portal venous, and equilibrium phases. Diffusion‐weighted images were acquired using an EPI sequence (TR/TE, 5600/60 ms; matrix, 128 × 128; slice thickness, 5 mm; intersection gap, 1.5 mm; field of view, 256 × 208 mm; with *b* values including 50 and 1000 s/mm^2^). Apparent diffusion coefficient (ADC) maps were calculated automatically from all diffusion weightings on a voxel‐by‐voxel basis. Follow‐up MRI was performed for assessing response to antifungal treatment. MR images were evaluated by two radiologists (H.W and H.Y., with 10 and 8 years of clinical experience, respectively) who were blinded to the clinical data, and final decisions were reached by consensus.

**Figure 2:**
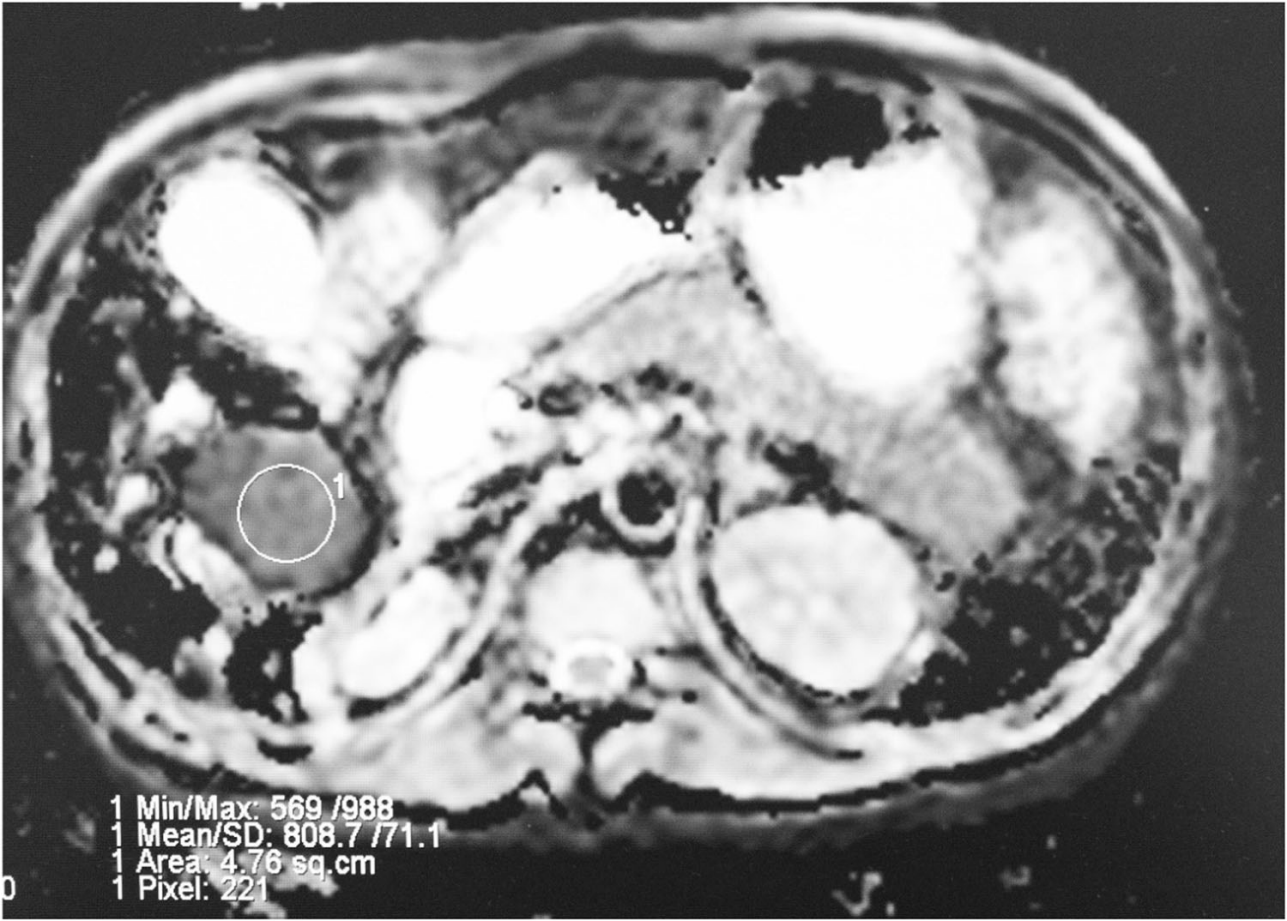
Illustration of ROI set on ADC map. ADC, apparent diffusion coefficient; ROI, region of interest.

The shape and signal intensities of the liver lesion were reviewed, and the size of the lesion was measured on T1‐weighted images, T2‐weighted images, and enhanced T1‐weighted images. The DWI data were postprocessed using workstation. The mean ADC values of the liver lesions of fungal infection and normal liver parenchyma were obtained by drawing manually regions of interest (ROI). One circular ROI was placed inside the lesion by adopting a manual single‐section ROI analysis (Figure 2). When the lesion was difficult to recognize on the ADC map, the T2‐weighted image and the contrast‐enhanced T1‐weighted images served as a roadmap for ROI placement.

### 2.4 Statistical analysis

The analyses were performed with statistical packages (SPSS 25.0; GraphPad Prism 9.1). Data for categorical variables are expressed as frequency rates and percentages. Continuous variables were described using the mean, median, and interquartile range (IQR). The mean ADCs of the hepatic fungal lesions and the normal liver parenchyma were compared with Student's *t*‐test. The mean ADCs of the hepatic fungal lesions of pretreatment and posttreatment were compared using paired *t*‐test. A receiver operating characteristic (ROC) curve analysis was used to define the ADC cutoff value for differentiating normal liver parenchyma from hepatic fungal infection lesions. Differences were considered significant at *p* < .05 with a two‐tailed test.

## 3 RESULTS

### 3.1 Patient characteristics

The demographic and clinical characteristics of the patients were shown in Table 1. A total of 13 patients (mean age, 31 years old) with hepatic fungal infections were enrolled in this study. Of these patients, seven patients (54%) presented with ALL, and six patients (46%) presented with AML. All patients (100%) presented with fever, and five (38%) presented with abdominal pain. Both the neutrophil count and white blood cell count were significantly lower than the normal range, whereas liver enzymes, alanine aminotransferase, and aspartate aminotransferase were within the normal range. Of 13 patients, fungal infections of three patients were proven by microbiology (*candida albicans* in two cases and aspergillus in one case), remaining 10 patients were diagnosed as probable fungal infection. Patients were treated with antifungal drugs (voriconazole, liposomal amphotericin B), ranging from 1–3 months.

**Table 1:** Baseline characteristics of the patients.

Parameter	Result
Number of patients	13
Age (years)	31 ± 13
Female	7 (54%)
Clinical diagnosis	
AML	6 (46%)
ALL	7 (54%)
Presenting symptoms	
Fever	13 (100%)
Abdominal pain	5 (38%)
Laboratory data	
White blood cell count (×10^9^/L)	2.66 ± 2.49
Neutrophil count (×10^9^/L)	1.45 ± 2.02
Alanine aminotransferase (U/L)	39.88 ± 37.00
Aspartate aminotransferase (U/L)	33.62 ± 38.63
Diagnosis based on EORTC‐MSG	
Proven	3 (23%)
Probable	6 (46%)
Possible	4 (31%)
Antifungal therapy effect assessed by MRI	
The lesions disappeared	5 (38%)
The lesions with various degree of absorbed	8 (62%)

*Note*: Data are expressed as the mean ± standard deviation or median (interquartile range) or *n* (%).

Abbreviations: AML, Acute myeloid leukemia; ALL, acute lymphoblastic leukemia; EORTC‐MSG; European Organization for Research and Treatment of Cancer and Mycoses Study Group. MRI, magnetic resonance imaging.

### 3.2 MRI for diagnosis of hepatic fungal infection

MRI of all the patients showed multiple lesions in the liver, and the lesions of four patients involved in spleen and the lesions of two patients involved in the kidneys.

**Figure 3:**
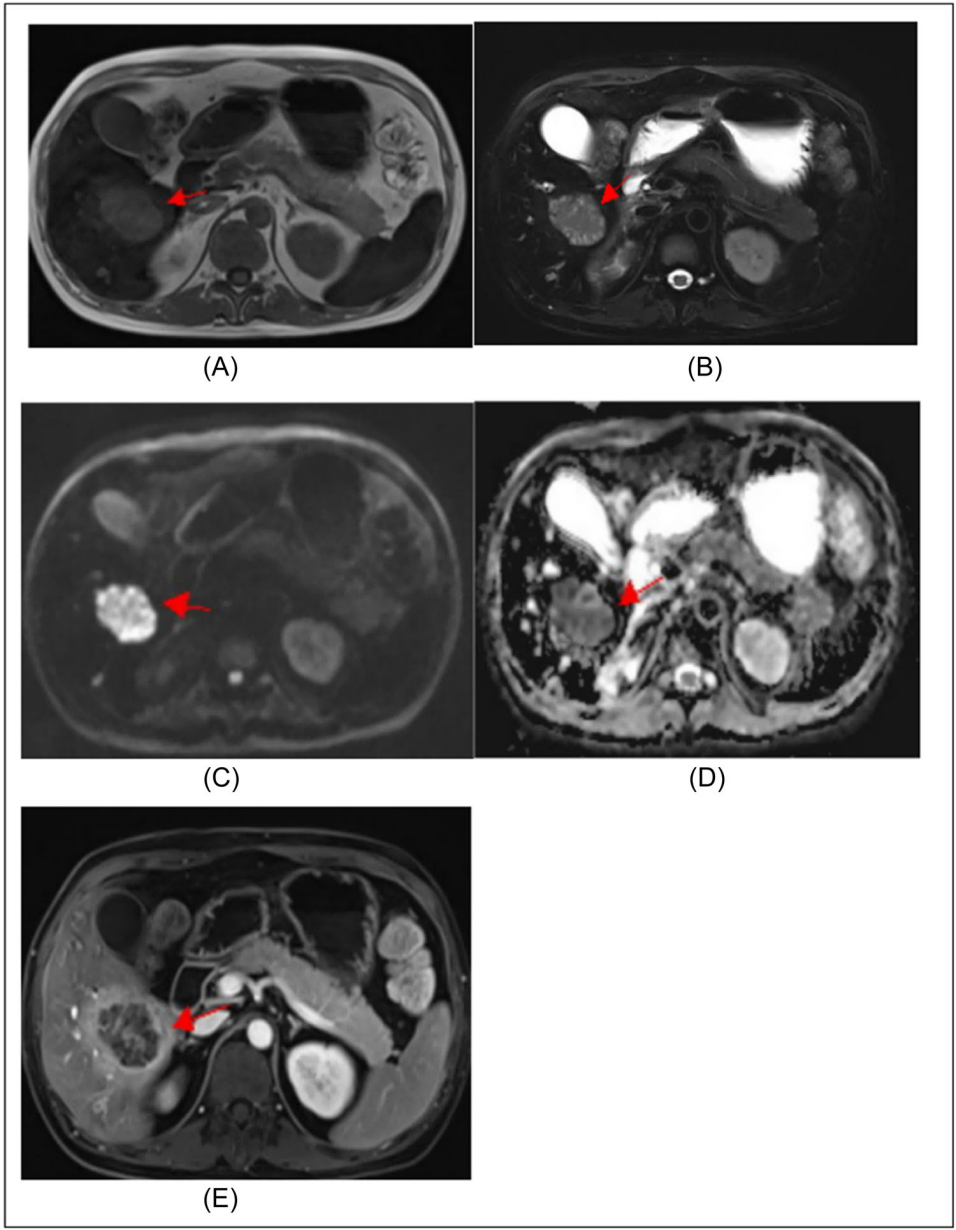
Representative MR images in a 30‐year‐old man patient with hepatic fungal infection. The images of the lesion on T1WI (A), T2WI (B), DWI (C), ADC map (D), and contrast enhancement (E).

**Figure 4:**
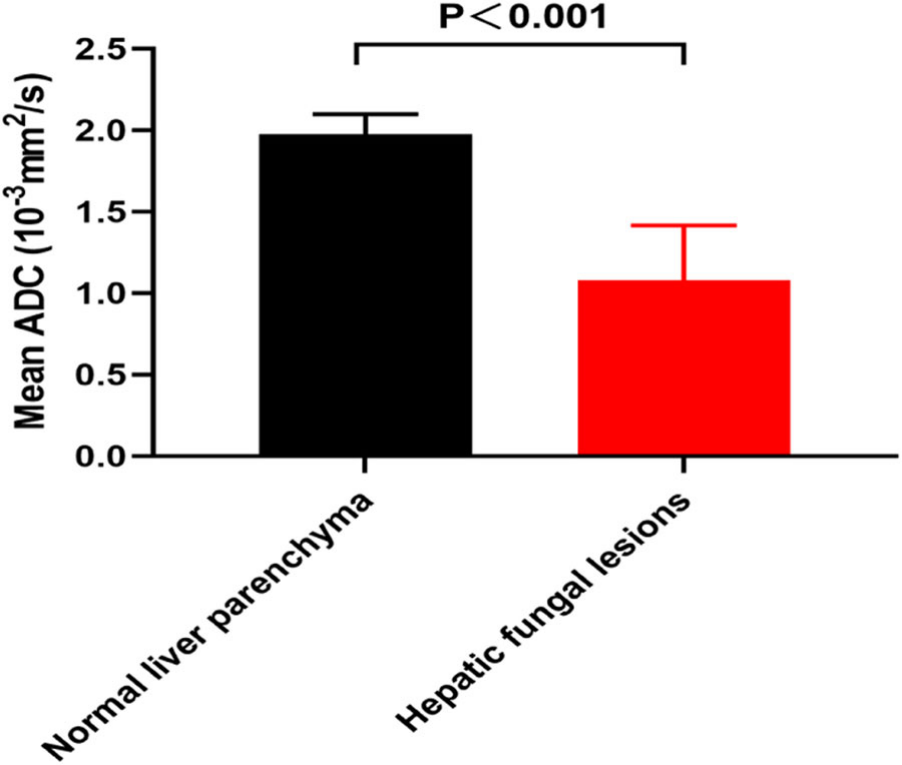
Graph (mean ± standard deviation) shows the results of the measured ADC values in the hepatic fungal lesions and normal liver parenchyma. ADC, apparent diffusion coefficient.

Lesions were rounded or oval‐shaped and measured from 0.3 to 3 cm in diameter. The lesions exhibited hyperintense signal on T2‐weighted images and iso‐hypointense signal on T1‐weighted images, with variable degrees of peripheral enhancement on gadolinium‐enhanced images. On functional MRI, the lesions showed a significantly hyperintense signal on DWI and a markedly hypointense signal on ADC maps, reflecting a marked restricted diffusion (Figure 3). Moreover, the mean ADC values of the lesions were significantly lower than those of normal liver parenchyma (1.08 ± 0.34 × 10^−3^ mm^2^/s vs. 1.98 ± 0.12 × 10^−3^ mm^2^/s, *p* < 0.001) (Figure 4).

### 3.3 MRI for differentiation of hepatic fungal infection

For the differentiation between normal liver parenchyma from hepatic fungal infection lesions, ROC curve analysis revealed that ADC measurements had a diagnostic accuracy, as given by the area under the curve, of 0.970 (95% confidence interval [CI]: 0.817–1.000) with a sensitivity of 92.3% and a specificity of 100% using a cutoff ADC value of 1.41 × 10^−3^ mm^2^/s.

### 3.4 MRI for treatment response assessment of hepatic fungal infection

Follow‐up MRI, with a median time of 50 days, was performed for assessing antifungal therapy response. After treatment, the lesions appeared hypointense to moderate hyperintense on T1‐weighted images and isointense to moderate hyperintense on T2‐weighted images. In addition, the lesions presented mild to moderate enhanced after gadolinium‐enhancement. The lesions disappeared in five cases and absorbed in eight cases with variable degree after therapy. Of these eight cases, ADC value in six cases could be measured for quantitatively evaluating the treatment response of hepatic fungal lesion.

**Figure 5:**
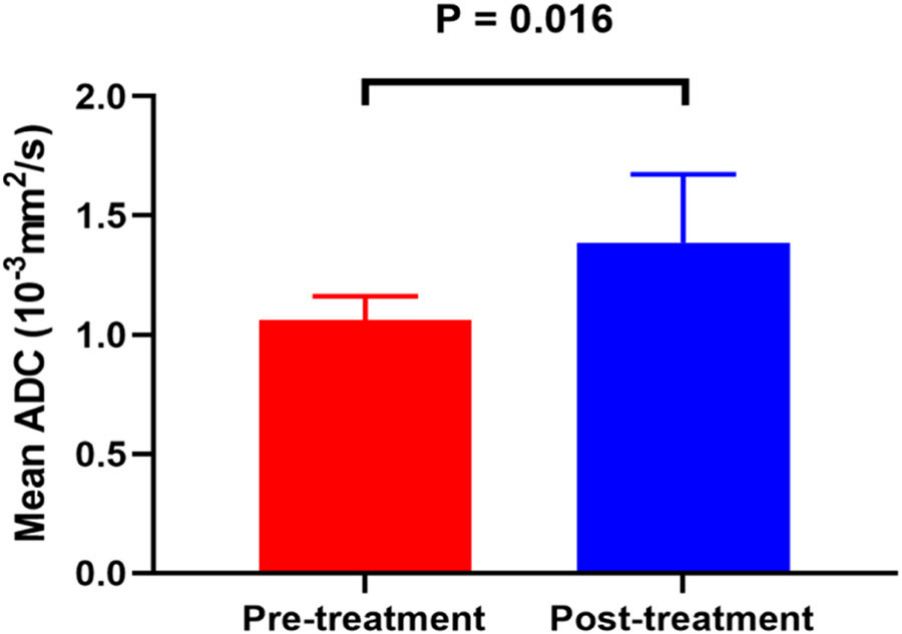
Graph (mean ± standard deviation) demonstrates the result of treatment response by assessing quantitatively the ADC values of the hepatic fungal lesions. ADC, apparent diffusion coefficient.

The mean ADC values of the lesions in posttreatment were significantly increased when comparing with those of pretreatment (1.39 ± 0.29 × 10^−3^ mm^2^/s vs. 1.06 ± 0.10 × 10^−3^ mm^2^/s, *p* = .016) (Figure 5).

## 4 DISCUSSION

In this study, we explored the value of DWI in diagnosis and therapy response assessment of hepatic fungal infection in patients with acute leukemia. We demonstrated that: 1) there were significant hyperintensities on DWI and hypointensities on ADC values in hepatic fungal lesions, when compared with normal liver parenchyma. 2) The mean ADC values of the hepatic fungal lesions in posttreatment were increased when comparing with pretreatment. To our best knowledge, our study was the first one to quantitatively evaluate diffusion features of hepatic fungal infection in patients with acute leukemia using DWI.

Invasive fungal infection was a serious complication in patients with hematologic malignancies and transplantation.^3^, ^5^, ^7^, ^9^, ^14^ *Candida*, *Aspergillus*, *Cryptococcus neoformans*, and *Histoplasma capsulatum* were the common pathogens of fungal infection. Lungs, liver, and spleen are the common deep organs of invasive fungal infection. Disseminated fungal disease could occur in 3%–29% of leukemia patients.^15^ In patients with acute leukemia, hepatic fungal infection was mainly caused by intensive chemotherapy or bone marrow transplant.^16^, ^17^, ^18^ In our study, all patients presented with fever despite apparently appropriate antibacterial therapy and neutropenia, the clinical presentations were consistent with previous studies.^4^, ^6^

Imaging played an important role in the diagnosis and evaluation of follow‐up of hepatic fungal infection in patients with acute leukemia. In our study, conventional MRI findings of hepatic fungal infection in patients with acute leukemia were consistent with previous studies.^9^, ^10^, ^11^, ^12^, ^19^ Importantly, we first explored the value of DWI for diagnosis and therapy response assessment of hepatic fungal infection.

Diffusion is the term used to describe the random (Brownian) motion of water molecules. DWI provides information of the mobility or viscosity of the water molecules in the tissue. We explored diffusion information of hepatic fungal infection using DWI. In this study, we found a marked hyperintensity on DWI and a hypointensity on ADC map in fungal hepatic abscesses in patients with acute leukemia, which could be attributed to the difference in the biochemical components of the necrotic contents. Tissues with different mobility or viscosity possess different ADC values. Hepatic abscess cavity is filled with pus, viscous fluid that consists of inflammatory cells, fungal components, and necrotic tissue with very high viscosity and cellularity, thereby resulting in significantly decreased ADC values. Thus, water diffusion in hepatic fungal infection is limited. Furthermore, we found that ADC measurements were significantly different between normal liver parenchyma from hepatic fungal infection lesions, as proved by ROC curve analysis.

Moreover, we quantitatively evaluate the therapy response of hepatic fungal infection with ADC value. We found that ADC values of lesions were gradually increased with persistent antifungal treatment, suggesting low viscosity of the lesion after treatment. Therefore, ADC measurement could be used to monitor the treatment effect in patients with acute leukemia.

There are some issues that need to be considered in our study. First, the sample size of this study was small, a large sample and longitudinal study need to be performed in the future. Second, this was a single‐center study, a center‐specific bias could not be excluded. However, advantages of single center study include constant work‐up routine and constant quality of MR examination.

## 5 CONCLUSIONS

DWI could provide diffusion information of hepatic fungal infection in patients with acute leukemia and could be taken as a valuable tool for diagnosis and therapy response assessment of these patients.

## AUTHOR CONTRIBUTIONS

**Haoyu Wang**: Conceptualization; writing—original draft. **Haitao Yu**: Formal analysis; writing—review & editing. **Dong Bai**: Software; writing—review & editing. **Dan Yao**: Writing—review & editing. **Yongjun Han**: Methodology; writing—review & editing. **Yichao Shi**: Conceptualization; writing—review & editing. **Zhiqun Wang**: Conceptualization; writing—review & editing.

## CONFLICT OF INTEREST STATEMENT

The authors declare no conflict of interest.

## Supporting information

Supporting information.

## References

[1] Gedik H , Yokuş O . Hepatosplenic candidiasis in patient with acute leukemia. Asian Pac J Trop Dis. 2015;5:S175‐S177.

[2] Fung JJ . Fungal infection in liver transplantation. Tranpl Infect Dis. 2002;4(suppl 3):18‐23. 10.1034/j.1399-3062.4.s3.3.x 12486788

[3] Rossetti F , Brawner DL , Bowden R , et al. Fungal liver infection in marrow transplant recipients: prevalence at autopsy, predisposing factors, and clinical features. Clin Infect Dis. 1995;20(4):801‐811. 7795077 10.1093/clinids/20.4.801

[4] Talbot GH . Persistent fever after recovery from granulocytopenia in acute leukemia. Arch Intern Med. 1988;148(1):129‐135. 3422147

[5] Celkan T , Kizilocak H , Evim M , et al. Hepatosplenic fungal infections in children with leukemia—risk factors and outcome: a multicentric study. J Pediatr Hematol Oncol. 2019;41(4):256‐260. 30730381 10.1097/MPH.0000000000001431

[6] Metser U , Haider MA , Dill‐Macky M , Atri M , Lockwood G , Minden M . Fungal liver infection in immunocompromised patients: depiction with multiphasic contrast‐enhanced helical CT. Radiology. 2005;235(1):97‐105. 15731367 10.1148/radiol.2351031210

[7] Shirkhoda A , Lopez‐Berestein G , Holbert JM , Luna MA . Hepatosplenic fungal infection: CT and pathologic evaluation after treatment with liposomal amphotericin B. Radiology. 1986;159(2):349‐353. 3961167 10.1148/radiology.159.2.3961167

[8] Benedetti NJ , Desser TS , Jeffrey RB . Imaging of hepatic infections. Ultrasound Q. 2008;24(4):267‐278. 19060716 10.1097/RUQ.0b013e31818e5981

[9] Karthaus M , Huebner G , Elser C , Geissler RG , Heil G , Ganser A . Early detection of chronic disseminated *Candida* infection in leukemia patients with febrile neutropenia: value of computer‐assisted serial ultrasound documentation. Ann Hematol. 1998;77(1):41‐45. 9760151 10.1007/s002770050409

[10] Anttila VJ , Lamminen AE , Bondestam S , et al. Magnetic resonance imaging is superior to computed tomography and ultrasonography in imaging infectious liver foci in acute leukaemia. Eur J Haematol. 1996;56(1‐2):82‐87. 8600000 10.1111/j.1600-0609.1996.tb00300.x

[11] Sallah S , Semelka R , Kelekis N , Worawattanakul S , Sallah W . Diagnosis and monitoring response to treatment of hepatosplenic candidiasis in patients with acute leukemia using magnetic resonance imaging. Acta Haematol. 1998;100(2):77‐81. 9792936 10.1159/000040869

[12] Kelekis NL , Semelka RC , Jeon HJ , Sallah AS , Shea TC , Woosley JT . Dark ring sign: finding in patients with fungal liver lesions and transfusional hemosiderosis undergoing treatment with antifungal antibiotics. Magn Reson Imaging. 1996;14(6):615‐618. 8897364 10.1016/0730-725x(96)00090-2

[13] Donnelly JP , Chen SC , Kauffman CA , et al. Revision and update of the consensus definitions of invasive fungal disease from the European Organization for Research and treatment of cancer and the Mycoses Study Group Education and Research Consortium. Clin Infect Dis. 2020;71(6):1367‐1376. 31802125 10.1093/cid/ciz1008 PMC7486838

[14] Verma A , Wade JJ , Cheeseman P , et al. Risk factors for fungal infection in paediatric liver transplant recipients. Pediatr Tranplant. 2005;9(2):220‐225. 10.1111/j.1399-3046.2005.00295.x 15787797

[15] Pagano L , Mele L , Fianchi L , et al. Chronic disseminated candidiasis in patients with hematologic malignancies. clinical features and outcome of 29 episodes. Haematologica. 2002;87(5):535‐541. 12010669

[16] Masood A , Sallah S . Chronic disseminated candidiasis in patients with acute leukemia: emphasis on diagnostic definition and treatment. Leuk Res. 2005;29(5):493‐501. 15755501 10.1016/j.leukres.2004.10.003

[17] Nicolato A , Nouér SA , Garnica M , Portugal R , Maiolino A , Nucci M . Invasive fungal diseases in patients with acute lymphoid leukemia. Leuk Lymphoma. 2016;57(9):2084‐2089. 26949001 10.3109/10428194.2016.1154957

[18] Kumar J , Singh A , Seth R , Xess I , Jana M , Kabra SK . Prevalence and predictors of invasive fungal infections in children with persistent febrile neutropenia treated for acute leukemia – a prospective study. Indian J Pediatr. 2018;85(12):1090‐1095. 29956075 10.1007/s12098-018-2722-0

[19] Semelka RC , Kelekis NL , Sallah S , Worawattanakul S , Ascher SM . Hepatosplenic fungal disease: diagnostic accuracy and spectrum of appearances on MR imaging. Am J Roentgenol. 1997;169(5):1311‐1316. 9353448 10.2214/ajr.169.5.9353448

